# Reliability of Quantitative Real-Time PCR for Bacterial Detection in Cystic Fibrosis Airway Specimens

**DOI:** 10.1371/journal.pone.0015101

**Published:** 2010-11-30

**Authors:** Edith T. Zemanick, Brandie D. Wagner, Scott D. Sagel, Mark J. Stevens, Frank J. Accurso, J. Kirk Harris

**Affiliations:** 1 Department of Pediatrics, University of Colorado Denver, Aurora, Colorado, United States of America; 2 Department of Biostatistics and Informatics, Colorado School of Public Health, University of Colorado Denver, Aurora, Colorado, United States of America; University of Alabama-Birmingham, United States of America

## Abstract

The cystic fibrosis (CF) airway microbiome is complex; polymicrobial infections are common, and the presence of fastidious bacteria including anaerobes make culture-based diagnosis challenging. Quantitative real-time PCR (qPCR) offers a culture-independent method for bacterial quantification that may improve diagnosis of CF airway infections; however, the reliability of qPCR applied to CF airway specimens is unknown. We sought to determine the reliability of nine specific bacterial qPCR assays (total bacteria, three typical CF pathogens, and five anaerobes) applied to CF airway specimens. Airway and salivary specimens from clinically stable pediatric CF subjects were collected. Quantitative PCR assay repeatability was determined using triplicate reactions. Split-sample measurements were performed to measure variability introduced by DNA extraction. Results from qPCR were compared to standard microbial culture for *Pseudomonas aeruginosa*, *Staphylococcus aureus*, and *Haemophilus influenzae*, common pathogens in CF. We obtained 84 sputa, 47 oropharyngeal and 27 salivary specimens from 16 pediatric subjects with CF. Quantitative PCR detected bacterial DNA in over 97% of specimens. All qPCR assays were highly reproducible at quantities ≥10^2^ rRNA gene copies/reaction with coefficient of variation less than 20% for over 99% of samples. There was also excellent agreement between samples processed in duplicate. Anaerobic bacteria were highly prevalent and were detected in mean quantities similar to that of typical CF pathogens. Compared to a composite gold standard, qPCR and culture had variable sensitivities for detection of *P. aeruginosa*, *S. aureus* and *H. influenzae* from CF airway samples. By reliably quantifying fastidious airway bacteria, qPCR may improve our understanding of polymicrobial CF lung infections, progression of lung disease and ultimately improve antimicrobial treatments.

## Introduction

Cystic fibrosis (CF) is a life-shortening, autosomal-recessive genetic condition. Lung disease characterized by chronic airway infection and inflammation is the leading cause of morbidity and mortality in patients with CF [1]. Airway infection is primarily attributed to a narrow spectrum of bacteria, most commonly *Pseudomonas aeruginosa* and *Staphylococcus aureus* [2], [3]. However, CF airway infections are frequently polymicrobial, and a broad range of bacteria including anaerobes likely contribute to CF lung disease [4]–[6]. In addition, airway cultures may not detect typical CF associated bacteria even during acute pulmonary exacerbations [7]. Reliance on standard microbial culture methods limits detection of polymicrobial infections, especially anaerobic bacteria, and the routine use of anaerobic culture is labor-intensive and unreliable [8]. Thus, the ability to study polymicrobial and anaerobic infection in CF has been limited.

Molecular detection techniques identify airway bacteria without reliance on culture [9], [10]. Nucleic acid amplification of 16S rRNA genes specific for the domain Bacteria followed by post-amplification analyses using terminal restriction fragment length polymorphism profiling (t-RFLP), Sanger sequencing, or pyrosequencing have been used to characterize the bacterial community in CF airway samples [11]–[14]. Results from these studies consistently find a high prevalence of anaerobic bacteria within a complex polymicrobial community. Quantitative real-time PCR (qPCR) employs primers specific for particular bacteria to detect and quantify bacteria without culture [15]. Quantitative PCR is increasingly used for bacterial identification in conditions such as sepsis, meningitis, vaginosis and gingivitis [16]–[20]. Possible applications of qPCR in CF include rapid detection and quantification of potentially pathogenic anaerobic bacteria, assessing response to antimicrobial therapy, and tracking longitudinal changes in airway microbiology in the clinic and in clinical trials. However, the reliability of qPCR applied to CF airway specimens, an important step in validating the use of qPCR in CF, has received little attention, particularly with respect to anaerobic bacteria.

The goals of this study were to determine the reliability of bacterial quantification from CF airway samples using qPCR and to compare qPCR results to standard culture for routinely cultured CF pathogens. We hypothesized that bacteria, including total bacteria, typical CF pathogens, and anaerobic bacteria, could be reproducibly quantified from CF airway samples using qPCR, and that qPCR would have comparable sensitivity and specificity to culture for detection of typical CF pathogens. To test this hypothesis, we applied qPCR to CF airway samples (oropharyngeal swab and sputum) to measure total bacteria; three typical CF pathogens, *Pseudomonas aeruginosa*, *Staphylococcus aureus*, and *Haemophilus influenzae*; and five anaerobic bacteria, *Prevotella melaninogenica*, *Prevotella oris*, *Prevotella denticola*, *Fusobacterium* species and *Peptostreptococcus micros*. We chose *P. aeruginosa*, *S. aureus*, *and H. influenzae* as bacterial targets as these are the most common airway pathogens detected in children with CF [21]. The panel of anaerobic bacteria was chosen based on preliminary 16S rRNA sequencing data from our laboratory showing that these bacteria are frequently present in CF airway samples during pulmonary exacerbations. Split-sample and triplicate measurements were performed to determine reproducibility of the qPCR assays. Salivary samples were also collected and DNA extracted; triplicate qPCR measurements were performed on the DNA extractions to determine intra-assay reproducibility. For *P. aeruginosa*, *S. aureus* and *H. influenzae*, qPCR and culture results were compared to determine sensitivity, specificity and correlation.

## Methods

### Ethics Statement

This study was approved by the Colorado Multiple Institutional Review Board (COMIRB). Written informed consent and HIPPA Authorization were obtained from all participants over the age of 17 years or from parents or legal guardians of participants younger than 18 years. Assent was obtained from all participants under 18 years.

### Study design

Eligible patients (age 8–21 years) were recruited from the CF clinic at The Children's Hospital Denver during routine CF clinic visits for this prospective study. Patients were identified as potential participants by the principal investigator and/or research coordinator prior to clinic visit, and approached by the research coordinator during the visit to determine patient interest. Inclusion criteria consisted of (1) a known diagnosis of CF based on sweat chloride >60 mEq/L or the presence of two known CF mutations and (2) clinically stable pulmonary disease as defined by clinical impression and forced expiratory volume in one second (FEV_1_) within 15% of baseline. Exclusion criteria consisted of (1) treatment with oral or intravenous antibiotics (excluding chronic azithromycin) in the thirty days prior to enrollment, intravenous or oral corticosteroids in the seven days prior to enrollment or non-steroidal anti-inflammatory medications in the three days prior to enrollment, and (2) FEV1 <40% predicted. Following enrollment, oropharyngeal, expectorated and induced sputa, and salivary specimens were collected from subjects at baseline, one month and one year visits. Oropharyngeal specimens were obtained by swabbing the posterior pharynx and bilateral tonsillar pillars with a sterile cotton swab. Prior to sputum collection, salivary specimens were collected into a sterile specimen cup using a published method by Navazesh [22]. Spontaneously expectorated sputum was collected in a sterile specimen cup. Sputum was induced by inhalation of 3% hypertonic saline as previously described [23].

### Standard CF bacterial culture

The clinical microbiology laboratory processed all airway specimens following standardized CF airway culture guidelines [2]. Oropharyngeal swabs were emulsified in 1 ml sterile saline. Sputa specimens were homogenized with 1% dithiothreitol (Calbiochem, Los Angeles, CA) in the presence of glass beads, and diluted with sterile saline (1∶10). Aliquots of each specimen were plated onto the following culture media: blood agar, MacConkey agar, *Haemophilus* isolation agar (HAE), *Burkholderia cepacia* selective agar (Remel Products, Lenexa, KS) and mannitol salt agar (MAN). Oropharyngeal culture results were semi-quantitative based on culture plate growth (range 1+ to 4+). *Pseudomonas aeruginosa* and other gram negative bacteria identified by sputum culture were reported and quantified if present in quantities ≥10^3^ colony-forming units (cfu) per milliliter. *Staphylococcus aureus* and *H. influenzae* colony counts were estimated from HAE and MAN plates. *Burkholderia cepacia* species identification was qualitative. Remaining specimen aliquots were frozen at −70 degrees Celsius for molecular studies. Salivary specimens were directly frozen without processing or culture at −70 degrees Celsius.

### Processing for molecular studies

Frozen oropharyngeal, sputa, and saliva specimens were thawed on ice. In order to examine variability in our DNA extraction process, split samples of oropharyngeal and sputa specimens were prepared and molecular analyses were run in parallel on the duplicate DNAs. Salivary specimens were not split, thus a single DNA extraction was performed. DNA was extracted from each sample directly using a modified bead beating and solvent extraction protocol [12]. Each sample was combined with 500 µl of 2x buffer B (143 mM Tris pH 8.0, 143 mM NaCl, 14 mM EDTA and 5.7% SDS), 500 µl phenol:chloroform, and approximately 0.25 g zirconium beads (0.1 mm, Biospec Products Inc.). The mixture was then mechanically disrupted by reciprocation for two minutes using an eight-channel bead beater (Biospec Products, Inc). Samples were centrifuged (16,000×g) for three minutes to separate the aqueous and organic phases. The DNA from 400 µl of the supernatant was precipitated using 7.5 M ammonium acetate (160 µl) and isoproponol (560 µl). This mixture was then centrifuged (16,000×g) for twenty minutes and decanted. The DNA pellet was washed with 1 ml of 70% ethanol and centrifuged for 5 minutes to ensure recovery of the DNA pellet. The DNA pellet was air dried, and re-suspended in 50 µl of TE (10 mM Tris pH 8.0, 1 mM EDTA). All extractions included a negative extraction control where buffer only was extracted in parallel with specimens. The precipitated DNA was used directly in qPCR reactions without further purification.

### Quantitative real-time PCR (qPCR)

Bacterial quantification was performed on DNA extracts using qPCR assays designed to measure (1) all bacteria, referred to as *bacterial load assay* [24] and (2) specific organisms, referred to as *specific organism assays*. Specific organism assays were performed for the following bacteria: *P. aeruginosa*, *S. aureus*, *H. influenzae*, *Prevotella melaninogenica*, *P. oris*, *P. denticola*, *Fusobacterium* species, and *Peptostreptococcus micros* [25]–[27]. All assays were performed by one of two technicians, trained in molecular diagnostics and blinded to the culture results. All qPCR assays were run in triplicate, and two negative PCR controls were run on each sample plate. All assays except for *H. influenzae* were based on previously published assays. The results are presented following Standards for Reporting of Diagnostic Accuracy (STARD) guidelines [28].

For *H. influenzae*, primers were designed from our in-house database containing hundreds of sequences obtained from CF airway specimens for *H. influenzae* with a product size of ∼600 base pairs. During development of the *H. influenzae* assay, we directly sequenced the amplicons from the first seven clinical samples that were positive for *H. influenzae* by qPCR. All amplicons were consistent with *H. influenzae* with ≥99% sequence identity (MegaBACE 1000 DNA Analysis System, GE Healthcare, Amerisham). To examine the specificity of our assay, we performed cross-reactivity studies by applying our qPCR assay to a three-fold dilution series (10^4^–10^6^) with two *H. influenzae* strains (ATCC49247 and ATCC10211) and three *H. parainfluenzae* strains (ATCC7901 and 2 patient strains from the clinical microbiology laboratory at The Children's Hospital Denver). In addition, three dilutions (10^4^–10^6^) with equal mixtures by weight of DNA from the two bacterial species and one 1∶100 *H. influenzae*: *H. parainflenzae* DNA mixture were analyzed with our *H. influenzae* qPCR assay. Results of qPCR were compared to the known amount of target bacterial DNA in each mixture.

#### Bacterial load assay

Total ribosomal RNA gene copy number was measured using a quantitative PCR (qPCR) TaqMAN assay (Roche Molecular Systems, Inc.), as described by Nadkarni and co-workers [24]. A cloned bacterial rRNA gene (*P. melaninogenica*) was used as the standard ranging in dilution from 10^2^ to 10^8^ copies on each plate. A clone with one 16S rRNA operon was used due to difficulty obtaining a precise copy number from genomic DNA and the predominance of slow-growing bacterium in CF airway samples [29], [30].

#### Specific organism assays

The qPCR reactions were run using the Power SYBR® Green PCR Master Mix (Applied Biosystems) and the appropriate PCR primers. Bacterial standards ranging in dilution from 10^2^ to 10^8^ copies were run on each plate. Melting temperature (Tm) was measured for each qPCR reaction. If the measured Tm was outside the prespecified range (determined from the standards) for each specific organism then the resulting copy number was considered not detected. Resulting values (Raw value, DNA copies/reaction) were log_10_ transformed. To confirm that the correct amplicon size was obtained, standards and reactions were analyzed by agarose gel electrophoresis.

Cross-reactivity studies were performed for *P. denticola*, *P. oris* and *P. melaninogenica* to insure specificity of the primers. *Fusobacterium* spp. was included as a control. Equal mixtures of the four bacteria of interest were created to total quantities ranging from 10^2^ to 10^8^ copies. Samples with *Fusobacterium* spp. standards were included on each plate. The assays for *P. denticola*, *P. oris* and *P. melaninogenica* were performed, and qPCR results were compared to the known amount of target bacterial DNA in each mixture.

Between-plate and within-plate studies were performed on a subgroup of airway specimens to measure precision and quantify sources of variability in the qPCR assays. Quantitative PCR assays for bacterial load and *P. melaninogenica* were performed on samples run on each of three plates and randomly assigned to three wells within a plate (9 replicates per sample).

### Quality Control Matrix

Results from all qPCR reactions were assessed using a quality control matrix designed to manage missing values and to identify unreliable results for repeat analysis. Coefficient of variation (CV) was calculated for each set of triplicate reactions. Results were considered consistent if all three values were not-detected, or if (1) all three replicates had detectable DNA, (2) the CV was less than 20%, and (3) the median value was less than 9. The upper limit of 10^9^ was set to identify any reaction with copy numbers outside the range of the standard curve. For samples with consistent results, the mean value of the three replicates was taken to represent the copies of bacterial rRNA genes present in the sample. If all three replicates were not-detected, then the quantity of bacteria present was assumed to be below the limit of detection. Inconsistent results were examined in more detail and in most cases the result was determined using a set of quality control rules developed by the authors. The following is an overview of those rules: (1) if two values were not-detected and the third value was <10 copies/reaction, then the sample value equaled the detected value, and (2) if two non-missing values were within 0.2 log, with the third value being either missing or different by more than 0.2 log, then the sample value equaled the mean of two values. Assays that did not meet these criteria were considered unacceptable and qPCR reactions were rerun. A more complete description of the specific quality control rules is provided in the supporting data (Figure S1).

### Statistical Analysis

Descriptive statistics include the mean and standard deviation or the median and 5^th^ and 95^th^ percentiles, where specified. The limit of detection for each assay was calculated as the mean plus three times the standard deviation of the measurement from negative controls. Paired t-tests were used to evaluate whether the split sample runs (B versus C) were significantly different. P-values <0.05 were considered statistically significant. Bland-Altman plots were used to visualize the agreement between split-sample runs. Additionally, comparisons between split sample measures were performed by categorizing whether or not the organism was present and calculating agreement between the two measurements. In order to examine our data for the secondary analysis, where random wells were assigned to standards and unknowns, we used a classical ANOVA model to explain the variation in the log-transformed data in terms of five factors (well, plate, duplicate, sample type and subject) and pairwise interactions within all the factors with exception of the well variable. Comparisons between two median values were performed using two-sample median tests. In order to determine the sensitivity of qPCR and culture for qualitative detection of standard CF pathogens (*P. aeruginosa*, *S. aureus* and *H. influenzae*), we created a composite “gold-standard” result. If either culture or qPCR was positive, then the composite result was positive. We then compared qPCR and culture to the composite results to determine their sensitivity. Specificity of qPCR was determined by comparing detection by qPCR using culture as the gold-standard. A non-parametric Spearman's rank correlation coefficient was assessed between the culture and qPCR measurements to compare quantitative results. All analyses were performed using SAS version 9.2 software (SAS Institute Inc.: Cary, NC, 2008).

## Results

### Specimens Collected

**Figure 1:**
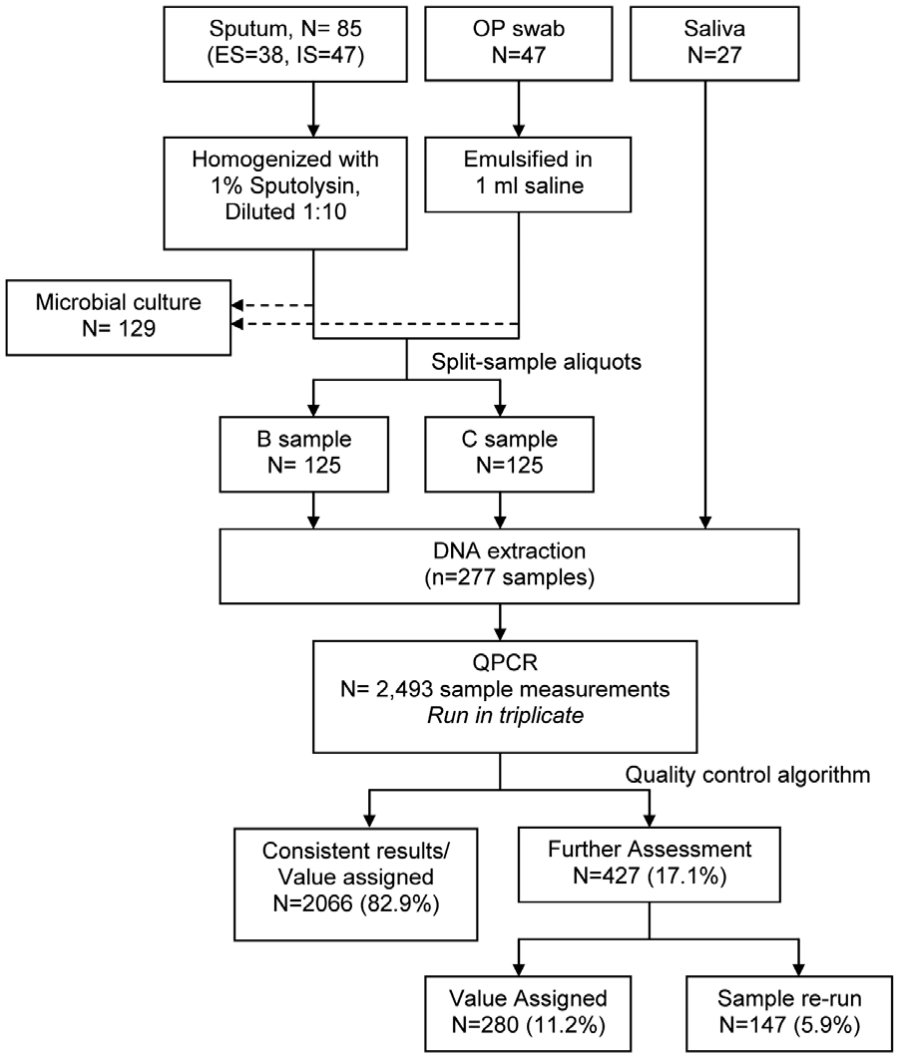
Specimen collection, processing, and quality control algorithm. Expectorated and induced sputa, oropharyngeal swabs and salivary specimens were collected and processed for standard CF microbial culture and/or DNA extraction (split-samples performed for sputa and oropharyngeal specimens). Bacterial load qPCR assay and eight specific organism qPCR assays were run in triplicate on all samples. Results were assessed using a quality control matrix and final value assigned to each sample. ES  =  expectorated sputum, IS  =  induced sputum, OP  =  oropharyngeal swab.

Eighteen CF subjects were enrolled between July 2006 and September 2007; the final study visit was completed in September 2008. Two subjects were withdrawn from the study due to the presence of pulmonary symptoms at the baseline visit. We collected 159 specimens from 16 subjects at three visits over one year consisting of 47 oropharyngeal swabs, 38 expectorated sputa, 47 induced sputa, and 27 saliva specimens. Subject characteristics at their baseline visit are shown in Table 1. No adverse events occurred during the study. Culture results were not obtained on saliva samples and in some cases specimens had inadequate quantities for both qPCR and culture. Seven specimens (six oropharyngeal and one expectorated sputum) underwent culture but not molecular analysis. Three specimens (one each of expectorated sputum, induced sputum and oropharyngeal swab) were analyzed only with molecular methods, resulting in 129 specimens with culture results, 152 specimens with qPCR results, and 122 specimens with both culture and qPCR. Specimen collection, processing and analysis are shown in Figure 1.

**Table 1:** Baseline subject characteristics (N = 16).

Characteristic	Value
Age, years, median (range)	13 (8–20)
Male gender, n (%)	10 (63)
Race	
Caucasian, n (%)	15 (94)
African-American, n (%)	1 (6)
Genotype	
F508del homozygous	7 (44)
F508del heterozygous	6 (38)
FEV_1_, percent predicted, median (range)	96 (58–133)
No. of specimens per patient, median (range)	10 (8–12)
Culture results from induced sputum, n (%)	
* Pseudomonas aeruginosa*	8 (50)
* Staphylococcus aureus*	9 (56)
* Haemophilus influenzae*	4 (25)

### Standard bacterial culture

The following bacteria were detected by standard CF bacterial culture from oropharyngeal and sputa specimens (n = 129 specimens): *S. aureus* (n = 81, 63%), *P. aeruginosa* (n = 58, 45%), *Streptococcus milleri* group (n = 26, 20%), *H. influenzae* (n = 17, 13%), *S. maltophilia* (n = 14, 11%) and *A. xylosoxidans*, (n = 10, 8%). Other bacteria detected infrequently consisted of: *Acinetobacter* spp. (n = 2), *Pseudomonas fluorescens* (n = 1), and *Streptococcus pyogenes* (n = 1). *Burkholderia cepacia* complex species were not detected from any specimen during the study.

### Quantitative real-time PCR

Split sample aliquots (referred to as B and C) from 125 oropharyngeal and sputa specimens were extracted in parallel and 27 saliva samples were extracted singly for a total of 277 DNA samples analyzed by qPCR (n = 2,493 sample measurements). For the bacterial load assay, bacterial rRNA genes were present in quantities ≥10^2^ copies/reaction in 97% of specimens including 98% of sputa, 95% of oropharyngeal and 100% of salivary specimens. For those samples with at least 100 copies of rRNA genes detected, the median quantity (log_10_ scale) was 4.9 copies/reaction (range 3.4–6.3). Results from all qPCR assays are shown in Table 2. Anaerobes were highly prevalent from all sample types with detection rates ranging from 20% (*P. oris*) to 73% (*P. melaninogenica*). When detected, anaerobes were present in quantities similar to that of CF-associated pathogens. The limits of detection for qPCR assays were as follows: bacterial load assay (n = 38 negative controls), 87 copies/reaction; *P. melaninogenica* (n = 28 controls), 5.1 copies/reaction; *H. influenzae* (n = 28 controls), 8.1 copies/reaction; and, *P. micros* (n = 30 controls), 38.7 copies/reaction. For all other assays, there were no DNA copies detected in any of the negative controls; thus, we could not calculate a limit of detection.

**Table 2:** Results from quantitative PCR assays.

qPCR assay (n = 277 samples)	Sample Type	Samples with mean ≥10^2^ copies/reaction, n (%)	Bacterial DNA not detected, n (%)	Acceptable value obtained after QC, n (%)	Samples rerun, n (%)	Median (5^th^–95^th^ percentile) log_10_ [copies/reaction] for samples with ≥10^2^ copies/reaction	CV for samples with ≥10^2^ copies/reaction, median (5^th^–95^th^ percentile)
Bacterial Load	All	269 (97)	7 (3)	273 (99)	4 (1)	4.9 (3.4 – 6.3)	2.1 (0.4 – 12.5)
	Sputum	164 (98)	4(2)	168 (100)	0 (0)	4.8 (3.4 – 6.1)	2.2 (0.5 – 9.8)
	OP	78 (95)	3 (4)	80 (98)	2 (2)	4.7 (3.1 – 5.7)	2.3 (0.4 – 13.2)
	Saliva	27 (100)	0 (0)	25 (93)	2 (7)	5.3 (4.1 – 6.6)	1.8 (0.4 – 17.0)
*P. aeruginosa*	All	63 (23)	161 (58)	269 (97)	8 (3)	3.2 (2.1 – 5.7)	2.9 (0.5 – 9.8)
	Sputum	56 (33)	81 (48)	162 (96)	6 (4)	3.2 (2.1 – 5.9)	2.9 (0.4 – 10.2)
	OP	3 (4)	61 (74)	80 (98)	2 (2)	3.3 (2.1 – 3.4)	3.3 (2.6 – 9.8)
	Saliva	4 (15)	19 (70)	27 (100)	0 (0)	2.9 (2.0 – 3.8)	4.6 (0.6 – 6.4)
*S. aureus*	All	62 (22)	192 (69)	265 (96)	12 (4)	3.4 (2.4 – 4.7)	5.4 (1.8 – 18.3)
	Sputum	55 (33)	102 (61)	160 (95)	8 (5)	3.4 (2.4 – 4.7)	5.3 (1.8 – 18.3)
	OP	1 (1)	74 (90)	79 (96)	3 (4)	2.2	8.6
	Saliva	6 (22)	16 (59)	26 (96)	1 (4)	2.9 (2.4 – 4.7)	5.5 (2.1 – 10.4)
*H. influenzae*	All	100 (36)	143 (52)	239 (86)	38 (14)	3.3 (2.3 – 4.2)	1.8 (0.3 – 7.3)
	Sputum	75 (45)	74 (44)	142 (85)	26 (15)	3.4 (2.3 – 4.3)	1.8 (0.3 – 7.3)
	OP	18 (22)	52 (63)	75 (92)	7 (9)	2.9 (2.7 – 3.5)	1.5 (0.1 – 7.0)
	Saliva	7 (26)	17 (63)	22 (82)	5 (19)	3.4 (2.3 – 3.6)	3.5 (1.0 – 15.7)
*P. melaninogenica*	All	203 (73)	12 (4)	268 (97)	9 (3)	3.2 (2.1 – 4.8)	2.9 (0.7 – 11.2)
	Sputum	122 (73)	5 (3)	162 (96)	6 (4)	3.3 (2.1 – 4.6)	3.4 (0.6 – 10.7)
	OP	60 (73)	6 (7)	80 (98)	2 (2)	2.9 (2.1 – 4.4)	2.9 (0.8 – 11.4)
	Saliva	21 (78)	1 (4)	26 (96)	1 (4)	4.0 (2.8 – 5.6)	1.7 (1.0 – 8.0)
*P. denticola*	All	72 (26)	89 (32)	266 (96)	11 (4)	2.8 (2.1 – 3.8)	3.8 (0.9 – 14.3)
	Sputum	44 (26)	61 (36)	162 (96)	6 (4)	2.9 (2.1 – 3.8)	3.4 (0.9 – 9.5)
	OP	13 (16)	22 (27)	77 (94)	5 (6)	2.6 (2.0 – 3.5)	5.7 (1.8 – 32.9)
	Saliva	15 (56)	6 (22)	27 (100)	0 (0)	2.7 (2.1 – 4.3)	2.4 (0.5 – 9.3)
*P. oris*	All	56 (20)	77 (28)	270 (98)	7 (3)	2.7 (2.1 – 4.2)	3.6 (0.6 – 15.3)
	Sputum	36 (21)	43 (26)	163 (96)	5 (3)	2.8 (2.1 – 4.2)	3.7 (0.6 – 16.2)
	OP	8 (10)	27 (33)	81 (99)	1 (1)	2.7 (2.2 – 2.9)	2.9 (0.8 – 14.0)
	Saliva	12 (44)	7 (26)	26 (96)	1 (4)	2.9 (2.0 – 4.0)	3.3 (0.4 – 6.3)
*F. nucleatum*	All	114 (41)	77 (28)	264 (95)	13 (5)	2.7 (2.1 – 4.1)	2.7 (0.6 – 7.6)
	Sputum	74 (44)	48 (29)	161 (96)	7 (4)	2.7 (2.0 – 4.0)	2.6 (0.6 – 7.6)
	OP	25 (30)	21 (26)	76 (93)	6 (7)	2.5 (2.1 – 4.1)	3.2 (0.7 – 9.7)
	Saliva	15 (56)	8 (30)	27 (100)	0 (0)	3.4 (2.2 – 5.0)	2.1 (0.3 – 6.0)
*P. micros*	All	183 (66)	21 (8)	232 (84)	45 (16)	3.0 (2.1 – 4.4)	3.4 (0.7 – 16.9)
	Sputum	107 (64)	9 (5)	133 (79)	35 (21)	3.1 (2.1 – 4.7)	3.1 (0.7 – 16.9)
	OP	52 (63)	11 (13)	75 (92)	7 (9)	2.9 (2.1 – 3.7)	4.7 (0.8 – 15.8)
	Saliva	24 (89)	1 (4)	24 (89)	3 (11)	3.3 (2.2 – 4.4)	2.7 (0.9 – 19.7)

**OP  =  Oropharyngeal swab; QC  =  quality control; CV  =  Coefficient of variation.**

### *H. influenzae* cross-reactivity studies

We examined DNA consensus data for close relatives of *H. influenzae* (Table S1). Our *H. influenzae* qPCR assay was applied to DNA samples from two ATCC strains of *H. influenzae* and three ATCC strains of *H. parainfluenzae*. After excluding copies amplified outside the Tm range set by the standard (87±2), there was no detectable bacterial DNA amplified in three-fold dilutions of *H. parainfluenzae* (n = 27 reactions). Both strains of *H. influenzae* amplified as expected with Tm ranging from 86–87 (n = 12 reactions). In mixture studies with a 1∶1 ratio by weight of DNA (n = 27 reactions), there was evidence of some cross-reactivity with mean amount of target bacterial DNA detected of 160% (range 107–268%). However, in mixture studies with 1∶100 ratio of *H. influenzae* to *H. parainfluenzae* (n = 9 reactions), the mean amount of target bacteria detected was 118% (range 98–140%) suggesting low levels of amplification of *H. parainfluenzae* (Tables S2 and S3).

### Reliability of qPCR

**Figure 2:**
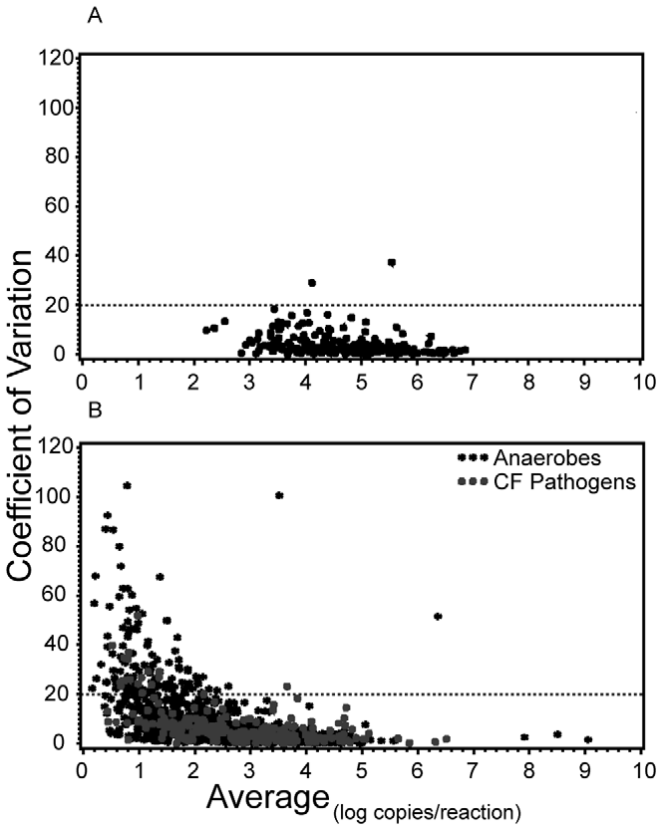
Relationship between qPCR reproducibility and quantity of bacterial DNA detected. Coefficient of Variation (CV) versus mean value of bacterial DNA (log_10_ copies/reaction) detected in triplicate reactions for each DNA sample measurement for (a) bacterial load assay, n = 277 sample measurements, and (b) specific organism assays, n = 2216 sample measurements.

For all assays, we compared the coefficient of variation (CV), calculated across the triplicates, to the average quantity (log_10_ copies per reaction) detected in each sample. Results for the bacterial load assay and specific organism assays are shown in Figures 2a and 2b, respectively. For samples with ≥2 log_10_ copies/reaction, all assays were highly reproducible with CV <20% for greater than 99% of samples. There was no difference in the repeatability of our assays by specimen type (oropharyngeal compared to sputum or saliva) or by specific organism assay. There was also no difference in repeatability between expectorated and induced sputum; thus, results from all sputa specimens are presented together. To confirm amplification of the correct PCR product, gel electrophoresis was performed, which confirmed that a single product was amplified.

### Comparison of split-samples

**Figure 3:**
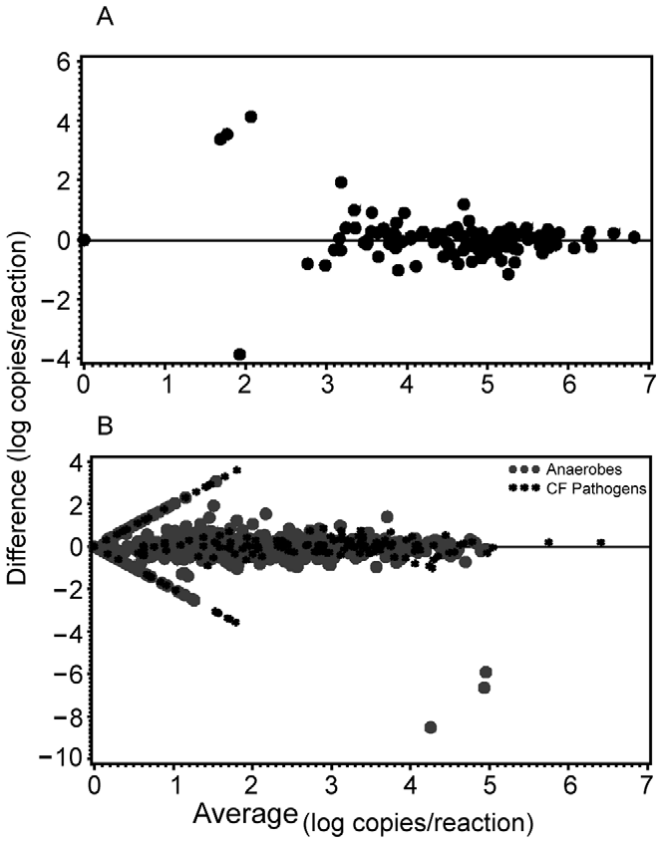
Comparison of qPCR assay results for split-sample measurements. Bland-Altman plots comparing split-sample measurements for (a) bacterial load assay (n = 125 paired sample measurements), and (b) specific organism assays (n = 1000 paired sample measurements). The straight lines indicate specimens with gene copies detected in one sample and not in the other matched pair.

We compared the B and C duplicates from oropharyngeal and sputa specimens (n = 125 DNA samples, 1125 sample measurements). The average difference between the duplicate runs was −0.02 log_10_ copies/reaction (95% CI: −0.06 – 0.03). There were no significant differences between duplicates when examined by assay and specimen type. There was 91% agreement between runs for all assays. By assay type, the agreement was as follows, bacterial load assay, 97%; *P. aeruginosa*, 92%; *S. aureus*, 93%; *H. influenzae*, 89%; *P. melaninogenica*, 94%; *P. oris*, 90%; *P. denticola*, 85%; *Fusobacterium* spp., 91%; and, *P. micros*, 86%. Bland-Altman plots are shown in Figures 3a and 3b which display the difference between the duplicate extractions versus the average for the bacterial load assay and specific organism assays respectively. The majority of points are evenly scattered near the line indicating a difference of zero, or equivalence. The median value of those samples that did not agree (bacteria present in one duplicate but not detected in the other) was 1.2 log_10_ copies/reaction (5^th^–95^th^ percentile range, 0.3–3.5) compared to 2.9 log_10_ copies/reaction (5^th^–95^th^ percentile range, 0.8–5.3) for those that did agree, indicating that the samples that did not agree had a lower values compared to those that did agree (p<0.01).

### Total precision and *Prevotella* cross-reactivity studies

Between-plate and within-plate studies were performed on 12 specimens consisting of eight sputa, two oropharyngeal, and four salivary specimens from four subjects. The sputa and oropharyngeal specimens were divided into B and C split-samples; thus, 24 DNA samples were analyzed. Each of the 24 samples was measured on each of three plates and was randomly assigned to three wells within a plate. Bacterial load and *P. melaninogenica* assays were performed on all samples (n = 432 sample measurements). The single largest source of variation was attributable to subject effects; this term provided by far the largest F-test value in the ANOVA. The lowest source of variability was between wells on a plate followed by plate-to-plate variability (Figure S2). There was less variability between subjects and specimen type in the all bacteria assay compared to the *P. melaninogenica* assay.

We performed cross-reactivity studies for *P. melaninogenica*, *P. oris*, and *P. denticola* assays. Between 10^3^ and 10^7^ copies, there was no evidence of cross-reactivity for any of the *Prevotella* qPCR assays with a mean amount of target bacteria detected of 91% (range, 73–113%) of the expected copy number. At the limits of the standard curve, 10^2^ and 10^8^ copies, there was evidence of cross-reactivity with a mean amount of target bacteria detected of 211% (range, 193–222%) (Table S4). For the *Fusobacterium* spp. standards, there was no bacterial DNA detected by any of the *Prevotella* assays.

### Quality Control Matrix

Overall, consistent results by triplicate qPCR assays were obtained for 82.9% of DNA sample measurements (n = 2,067/2,493) including 31.3% of sample measurements that had no target bacterial DNA detected (n = 780). Using our quality control (QC) rules, we determined a value for 11.2% of remaining sample measurements (n = 279), and only 5.9% of sample measurements (n = 147) were rerun. The majority of sample measurements identified as inconsistent had final values less than 100 copies/reaction (n = 324 of 427, 76%), suggesting more assay variability at low quantities.

The subset of 427 sample measurements (17.1%) that were considered inconsistent but assigned values either by the QC rules or after re-running the assay were used to compare the results of using the QC rules versus simply averaging the three triplicate values. The majority of sample measurements (n = 272 of 427, 64%) were assigned equivalent values using either the QC rules or simply averaging. There were 134 sputa or oropharyngeal specimens where at least one of the duplicate extractions was considered inconsistent and where the final values assigned differed across the two approaches. There was no striking improvement in the comparison of the duplicate extractions using the QC rules when looking across all samples; however, for those samples where the difference between the duplicates was ≥10^2^ copies/reaction (n = 25 sample measurements), the use of the QC rules yielded better results with less difference between B and C samples in 60% of sample measurements (n = 15) compared to simply averaging the triplicate values.

### Comparison of qPCR and microbial culture

We compared results from qPCR with microbial culture for *P. aeruginosa*, *S. aureus* and *H. influenzae*. For sputa specimens, culture and qPCR results were congruent in 90% for *P. aeruginosa*, 78% for *S. aureus*, and 54% for *H. influenzae*. For oropharyngeal swab samples, culture and qPCR results were congruent in 75% for *P. aeruginosa*, 35% for *S. aureus*, and 75% for *H. influenzae*. Among all sample types, discordant results consisted of 10 culture positive/qPCR negative and 8 qPCR positive/culture negative for *P. aeruginosa*, 43 culture positive/qPCR negative and 1 qPCR positive/culture negative for *S. aureus*, and 6 culture positive/qPCR negative and 42 qPCR positive/culture negative for *H. influenzae*. (Table 3) For *S. aureus*, we examined the quantity detected by quantitative culture for sputa specimens with discordant results. For those that were detected in culture, the median quantity in the samples missed by qPCR was 7.9×10^3^ cfu/ml (IQR, 2.0×10^3^ – 7.9×10^4^) which was lower than for those detected by qPCR [4.0×10^6^ cfu/ml (IQR, 1.0×10^5^–2.0×10^7^), p<0.01].

**Table 3:** Cross-tabulation of qPCR and standard microbial culture results for typical CF pathogens (N = 122 airway specimens consisting of 82 sputa and 40 oropharyngeal specimens).

	qPCR Negative/Culture Negative	qPCR Negative/Culture Positive	qPCR Positive/Culture Negative	qPCR Positive/Culture Positive
***P. aeruginosa***	58	10	8	46
Sputum	36	2	6	38
Oropharyngeal	22	8	2	8
***S. aureus***	42	43	1	36
Sputum	32	18	0	32
Oropharyngeal	10	25	1	4
***H. influenzae***	63	6	42	11
Sputum	36	4	34	8
Oropharyngeal	27	2	8	3

**Figure 4:**
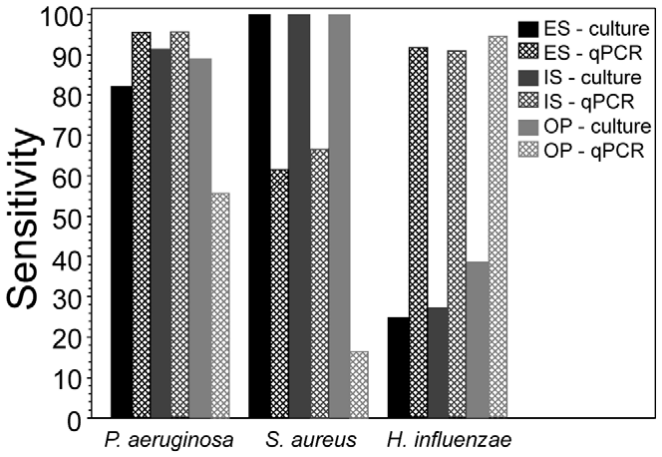
Sensitivity of qPCR and culture compared to a composite gold-standard for *P. aeruginosa*, *S. aureus*, and *H. influenzae*. (N = 122 specimens) ES =  expectorated sputum, IS =  induced sputum, OP =  oropharyngeal swab.

The sensitivity of qPCR assays and culture compared to the composite gold standards are shown in Figure 4. Culture and qPCR had similar sensitivities for detection of *P. aeruginosa* from sputum, but qPCR was less sensitive from oropharyngeal swabs. Culture was more sensitive for *S. aureus*, especially from oropharyngeal swabs, and less sensitive for *H. influenzae* compared to qPCR. Using culture as the gold standard, the sensitivity and specificity of qPCR was generally higher in sputum samples versus throat swab and was best for *P. aeruginosa*. The correlation between quantitative culture and qPCR for sputa samples (n = 82) for *P. aeruginosa* was 0.82 (p<0.01), for *S. aureus* was 0.63 (p<0.01) and for *H. influenzae* was 0.23 (p<0.04). To further examine the low sensitivity of the *S. aureus* qPCR assay, we determined the sensitivity of qPCR compared to culture relative to quantitative culture results. For specimens with *S. aureus* present in quantities between 10^3^–10^5^ cfu/ml by culture (n = 18), the sensitivity of qPCR was 22%. For specimens with *S. aureus* present in quantities >10^5^ cfu/ml by culture (n = 31), the sensitivity of qPCR was 87%. (Figure S3)

## Discussion

Our results indicate that bacterial DNA, including all bacteria, typical CF pathogens, and anaerobic bacteria, can be reproducibly quantified from CF airway specimens using qPCR. The bacterial load assay and specific organism assays had high intra-assay repeatability for specimens with rRNA gene copy numbers ≥10^2^. In addition, qPCR results for split samples had excellent agreement for bacterial DNA detection and quantification indicating that little variability was introduced by our DNA extraction technique. Results from between-plate and within-plate analyses also indicate that variability in sample measurements was primarily attributable to subject and specimen differences and that processing variability was negligible. Anaerobes were detected frequently by qPCR from all airway specimen types.

Our results also indicate that the qPCR assays for *P. aeruginosa*, *S. aureus* and *H. influenzae* have variable sensitivity compared to culture for bacterial detection from CF airway specimens. Quantitative PCR and culture had similar sensitivity for detection of *P. aeruginosa* from sputa, but qPCR was less sensitive for detection of *P. aeruginosa* from oropharyngeal specimens. *Staphylococcus aureus* was detected less frequently by qPCR, particularly from oropharyngeal swabs and from sputum specimens with low quantities of *S. aureus* by quantitative culture (<10^5^ cfu/ml). The increased sensitivity from sputum, especially for specimens with higher bacterial counts, and the high agreement between split-samples despite overall low sensitivity suggests that the limitation of qPCR is related to specimen characteristics, particularly low quantities of bacterial DNA, rather than inconsistency in assay performance. Unlike *P. aeruginosa* and *S. aureus*, *H. influenzae* was detected more often by qPCR than by culture from all specimen types.

The ability to reproducibly quantify anaerobic bacteria from CF airway samples is an important advance in studying the complex CF airway microbiome. Two important developments sparked recent interest in anaerobic infection in CF. First, Worlitzsch and co-workers demonstrated that *P. aeruginosa* was found in hypoxic mucus plugs within the CF airway lumen in patients with chronically infected with *P. aeruginosa* [6]. Second was the development of molecular techniques that allow detection of fastidious bacteria including anaerobic bacteria without the need for culture [9], [31], [32]. Multiple studies using molecular techniques or strict anoxic culture have demonstrated anaerobic bacteria in CF airway samples, including during pulmonary exacerbations [4], [5], [12], [14], [33], [34]. Studies using molecular detection have relied primarily on PCR amplification with universal bacterial primers followed by terminal restriction fragment length polymorphism (t-RFLP) analysis or sequencing. These methods provide an important overview of the microbial community present in each sample. Unlike microbial community analysis, qPCR allows quantification of specific bacteria of interest. Potential applications of qPCR to CF include rapid detection and quantification of potentially pathogenic anaerobic bacteria, assessment of response to antimicrobial therapy, and tracking longitudinal changes in airway microbiology.

There are several potential limitations to our study and the use of qPCR in CF. As oropharyngeal swabs are the primary means of non-invasive bacterial surveillance in young children with CF, the low sensitivity of these qPCR assays for *P. aeruginosa* and *S. aureus* may limit the utility of qPCR in this population [35]. Unlike our findings, Matsuda and co-workers reported excellent sensitivity of qPCR compared to culture for detection of these bacteria from blood specimens using these primers. There are several possible explanations for our lower sensitivity. CF sputum is highly tenacious and typically contains a large amount of inflammatory cells, primarily neutrophils resulting in release of large amounts of human DNA which may interfere with qPCR detection of relatively small quantities of bacterial DNA[36]. Second, *S. aureus* bacterium may not have been sufficiently lysed by our processing methods. However, specimens were processed twice with bead-beating techniques prior to qPCR so help ensure adequate lysis. Also, there was excellent agreement between parallel DNA extractions suggesting that lysis was at least consistent between samples. Third, bacterial DNA may have been present below the level of detection for qPCR. To investigate this further, we determined the sensitivity of qPCR relative to quantitative culture results and found significantly higher sensitivity in specimens with ≥10^5^ cfu/ml. As oropharyngeal swabs may have lower quantities of bacterial DNA than sputa samples, the finding of lower sensitivity for oropharyngeal specimens for both *P. aeruginosa* and *S. aureus* supports this explanation. Finally, the growth phase of bacteria or the presence of biofilms also may impact the quantity and detection of rRNA genes [25], [29], [37].

Another limitation to our study was the lack of anaerobic culture with which to compare our qPCR assays for anaerobic bacteria. Anaerobic cultures of airway samples are problematic in that they are labor intensive, outside standard practice for most clinical microbiologic laboratories and may have variable performance; thus, their utility as a gold-standard comparison is limited. To address this concern, we analyzed our qPCR products using gel electrophoresis to identify side products that may have interfered with our results and performed cross-reactivity studies for the *Prevotella* assays. We found minimal cross-reactivity among *Prevotella* species except at extreme low and high levels of bacterial DNA. The qPCR assays used for *P. micros* and *Fusobacterium* are published assays that have been shown to have good agreement with anaerobic culture from periodontal samples with minimal cross-reactivity with other bacterial species [27].

Unlike our findings with *S. aureus* and *P. aeruginosa*, qPCR appears more sensitive than culture for detection of *H. influenzae.* Dithiothreitol, used for sputum homogenization as recommended in CF airway culture guidelines, has been shown to inhibit the growth of *H. influenzae* in culture [2], [38]. Although the use of dithiothreitol may in part explain the low sensitivity of sputum culture compared with qPCR, oropharyngeal specimens not processed with dithiothreitol also had a low detection rate of *H. influenzae* by culture. In findings similar to our results, Van Belkum and colleagues detected *H. influenzae* from 4 of 6 CF untreated sputa samples by PCR but not by culture using PCR amplification of bacterial small subunit rDNA followed by probe mediated bacterial identification [39]. Thus, PCR may be more sensitive than culture for *H. influenzae*. To examine the specificity of the qPCR assay for *H. influenzae*, sequencing was performed on a sub-group of assays. The amplified PCR product was confirmed as *H. influenzae* with ≥99% sequence identity. We found some cross-reactivity between *H. influenzae* and *H. parainfluenzae* in mixed reactions, but in the absence of *H. influenzae*, there was no amplification of *H. parainfluenzae* within the Tm range determined by standards.

We also explored the use of a quality control matrix designed to manage missing values and to identify unreliable results for repeat analysis. Overall, less than 1% of sample measurements had improved agreement between split-sample measurements with the application of our QC matrix. For the large majority of our samples, simple averaging of triplicate measurements (with missing values set to not-detected) resulted in equivalent values and agreement between split-samples as our results with the application of QC.

In conclusion, we found that qPCR is a reproducible method for detection of bacteria including anaerobic bacteria from CF airway samples. Using qPCR, anaerobes were detected frequently from CF airway specimens, in quantities similar to that of typical CF associated pathogens. The sensitivity of our qPCR assays for detection of *P. aeruginosa* and *S. aureus*, especially from oropharyngeal specimens may limit its usefulness for detection of these pathogens. Refinement of these qPCR assays, including the use of alternative primers, may help address this limitation. The application of qPCR to airway specimens may improve our understanding of the clinical importance of polymicrobial infection and anaerobic bacteria, progression of lung disease and ultimately improve antimicrobial treatments in CF.

## Supporting Information

Table S1 **Comparison of DNA priming sites for** ***H. influenzae*** **and selected relatives.**
(PDF)

Table S2 *H. influenzae* qPCR assay specificity studies.
(DOC)

Table S3 ***H. influenzae*** **cross-reactivity studies.**
(DOC)

Table S4 ***Prevotella*** **cross-reactivity data.** Equal mixtures of *P. melanogenica*, *P. oris*, *P. denticola* and *F. nucleatum* were analyzed with qPCR assays for *Prevotella* spp.. *Fusobacterium nucleatum* standards were run on all plates.
(XLS)

Figure S1 **Quality control matrix with decision tree for samples with inconsistent results.** There were 2,493 sample measurements (277 DNA samples x 9 qPCR assays) performed in triplicate. Coefficient of variation (CV) was calculated for each set of triplicate reactions. Results were considered consistent if all three values were not-detected, or if (1) all three replicates had detectable DNA, (2) the CV was less than 20%, and (3) the median value was less than 9. The upper limit of 10^9^ was set to identify any reaction with copy numbers outside the range of the standard curve. For samples with consistent results, the mean value of the three replicates was taken to represent the copies of bacterial rRNA genes present in the sample. If all three replicates were not-detected, then the quantity of bacteria present was assumed to be below the limit of detection. Inconsistent results were examined in more detail and in most cases the result was determined using a set of quality control rules as follows: (1) if two values were not-detected and the third value was <10 copies/reaction, then the sample value equaled the detected value, and (2) if two non-missing values were within 0.2 log, with the third value being either missing or different by more than 0.2 log, then the sample value equaled the mean of two values. Assays that did not meet these criteria were considered unacceptable and qPCR reactions were rerun.
(TIF)

Figure S2 **ANOVA analysis of total precision with between-run and within-run comparisons.** Subject was the primary factor contributing to differences in measurement across plates and across wells (n = 24 samples). Five factors and six pairwise interactions were analyzed. There was greater variability across subjects for *P. melaninogenica* than for bacterial load assay.
(TIF)

Figure S3 **Sensitivity of** ***S. aureus*** **qPCR assay is highest for samples with quantities of** ***S. aureus*** **≥10^5^ cfu/ml by culture (n = 49 specimens).**
(TIF)
