# Supplementary material for: Reliability of Quantitative Real-Time PCR for Bacterial Detection in Cystic Fibrosis Airway Specimens
Source: PLoS One. 2010 Nov 30;5(11):e15101. doi: 10.1371/journal.pone.0015101 (PMC2994853; doi:10.1371/journal.pone.0015101)
Supplement: Table S1 — Comparison of DNA priming sites for H. influenzae and selected relatives. (PDF) [file pone.0015101.s001.pdf]

Table S1. Comparison of DNA priming sites for *H. influenzae* and selected relatives

Haemophilus influenzae (M35019)	CGGAAGATGAAAGTGCGG	...	GGGGTTGGGGTTTAACTC
Haemophilus pittmaniae (AJ290755)	***************T**	...	***A*****C**A*TG**
Pasteurella pneumotropica (AY362924)	*TA*G**CA****G*-**	...	*********C*****G**
Haemophilus parainfluenzae (EU083530)	**A************T**	...	*******A*C*****GCT
Haemophilus aegyptius (AY362905)	**AG***C**********	...	******************
Aggregatibacter actinomycetemcomitans (M75039)	***G***********T**	...	***A**********G*C*
Aggregatibacter aphrophilus (AY362906)	***G***********T**	...	***A***U*C***G*GCT
*Aggregatibacter segnis (*M75043)	**AG***C**********	...	***A*****CC**G*GCT
Actinobacillus seminis (AY362897)	***G*********G*T**	...	***A*****C*A***GCT
Pasteurella mairii (AY362923)	***G***********T**	...	***A*****C*****G*T
Pasteurella aerogenes (U66491)	**AG***C*****AC***	...	***A*****C*****GCT
Haemophilus haemoglobinophilus (AY362907)	********T****ACT**	...	***A*****C***G*GCT
Actinobacillus indolicus (AY362891)	**A****CG****G*T**	...	***A*****C***GTGCT
Haemophilus parasuis (AY362909)	***G*********ACT**	...	***A*****C**AG*GCT
Actinobacillus delphinicola (AY362889)	**AG****T******T**	...	***A*****C****GGT*
Actinobacillus scotiae (Y09653)	**AG****T*G****T**	...	*********CAA***GCT
Actinobacillus rossii (AF025839)	***G***********T**	...	***A***A*C*****GCT
Pasteurella langaaensis (AY362922)	*************GCT**	...	*******A*C**A**GCT
Actinobacillus porcinus (U65583)	*U*************T**	...	***A*****C***N*GCT
