# Supplementary material for: Reliability of Quantitative Real-Time PCR for Bacterial Detection in Cystic Fibrosis Airway Specimens
Source: PLoS One. 2010 Nov 30;5(11):e15101. doi: 10.1371/journal.pone.0015101 (PMC2994853; doi:10.1371/journal.pone.0015101)
Supplement: Table S2 — H. influenzae qPCR assay specificity studies. (DOC) [file pone.0015101.s002.doc]

Table S2. *H. influenzae* qPCR assay specificity studies.

| Bacterial strain | Concentration (nanograms) | Reactions (N) | Tm | Average rDNA gene copies/reaction detected* |
| --- | --- | --- | --- | --- |
| Hin (ATCC 49247) | 0.25 | 3 | 86 | 7.1E+06 |
| Hin (ATCC 49247) | 0.025 | 3 | 86 | 5.7E+05 |
| Hin (ATCC 49247) | 0.0025 | 3 | 86-87 | 5.1E+04 |
| Hin (ATCC 10211) | 0.25 | 3 | 86 | 5.2E+06 |
| Hpa (ATCC 7901) | 0.25 | 3 | 61-81 | ND |
| Hpa (ATCC 7901) | 0.025 | 3 | 70-82 | ND |
| Hpa (ATCC 7901) | 0.0025 | 3 | 70 | ND |
| Hpa (PS 1) | 0.25 | 3 | 69-87 | ND |
| Hpa (PS 1) | 0.025 | 3 | 69-70 | ND |
| Hpa (PS 1) | 0.0025 | 3 | 60-70 | ND |
| Hpa (PS 2) | 0.25 | 3 | 56-77 | ND |
| Hpa (PS 2) | 0.025 | 3 | 71-78 | ND |
| Hpa (PS 2) | 0.0025 | 3 | 72-78 | ND |

Hin= *H. influenzae*; Hpa=*H. parainfluenzae*; ATCC= American Type Culture Collection; PS= patient strain; ND= not detected; Tm = melting temperature; Tm range for *H. influenzae* based on standards = 85-89; * If measured Tm was outside the prespecified range then the resulting copy number was considered not detected.
