# Supplementary material for: Reliability of Quantitative Real-Time PCR for Bacterial Detection in Cystic Fibrosis Airway Specimens
Source: PLoS One. 2010 Nov 30;5(11):e15101. doi: 10.1371/journal.pone.0015101 (PMC2994853; doi:10.1371/journal.pone.0015101)
Supplement: Table S3 — H. influenzae cross-reactivity studies. (DOC) [file pone.0015101.s003.doc]

Table S3. *H. influenzae* cross-reactivity studies

| Bacterial strains | Ratio | Concentration (nanograms) | Reactions (N) | Tm | Expected rDNA gene copies/ reaction (mean)† | Detected rDNA gene copies/ reaction (mean) | % Target bacterial DNA detected |
| --- | --- | --- | --- | --- | --- | --- | --- |
| Hin1: Hpa1 | 1:1 | 0.25 | 3 | 86-87 | 3.5E+06 | 4.2E+06 | 120% |
| Hin1:Hpa2 | 1:1 | 0.25 | 3 | 86 | 3.5E+06 | 4.1E+06 | 117% |
| Hin2:Hpa3 | 1:1 | 0.25 | 2* | 86 | 2.6E+06 | 2.8E+06 | 107% |
| Hin1: Hpa1 | 1:1 | 0.025 | 3 | 86-87 | 2.9E+05 | 4.9E+05 | 172% |
| Hin1:Hpa2 | 1:1 | 0.025 | 3 | 86-87 | 2.9E+05 | 4.8E+05 | 164% |
| Hin2:Hpa3 | 1:1 | 0.025 | 3 | 86 | 2.9E+05 | 7.8E+05 | 268% |
| Hin1: Hpa1 | 1:1 | 0.0025 | 2* | 86 | 2.5E+04 | 5.1E+04 | 201% |
| Hin1:Hpa2 | 1:1 | 0.0025 | 3 | 86-87 | 2.5E+04 | 4.3E+04 | 170% |
| Hin2:Hpa3 | 1:1 | 0.0025 | 3 | 86 | 2.5E+04 | 3.1E+04 | 124% |
| Hin2:Hpa1 | 1:100 | 0.25 | 3 | 86-87 | 2.5E+04 | 3.5E+04 | 140% |
| Hin2:Hpa2 | 1:100 | 0.25 | 2* | 86 | 2.5E+04 | 2.5E+04 | 98% |
| Hin2:Hpa3 | 1:100 | 0.25 | 3 | 86 | 2.5E+04 | 2.9E+04 | 117% |

Hin= *H. influenzae*; Hpa= *H. parainfluenzae*; Hin1= ATCC 49247 strain; Hin2= ATCC 10211 strain; Hpa1= ATCC 7901 strain; Hpa2= patient strain 1; Hpa3= patient strain 2; Tm= melting temperature, * Indicates one reaction failed to amplify DNA, † Expected copy numbers calculated from data in Supporting Data Table 2.
