# Supplementary figures and images for: Reliability of Quantitative Real-Time PCR for Bacterial Detection in Cystic Fibrosis Airway Specimens
Source: PLoS One. 2010 Nov 30;5(11):e15101. doi: 10.1371/journal.pone.0015101 (PMC2994853; doi:10.1371/journal.pone.0015101)

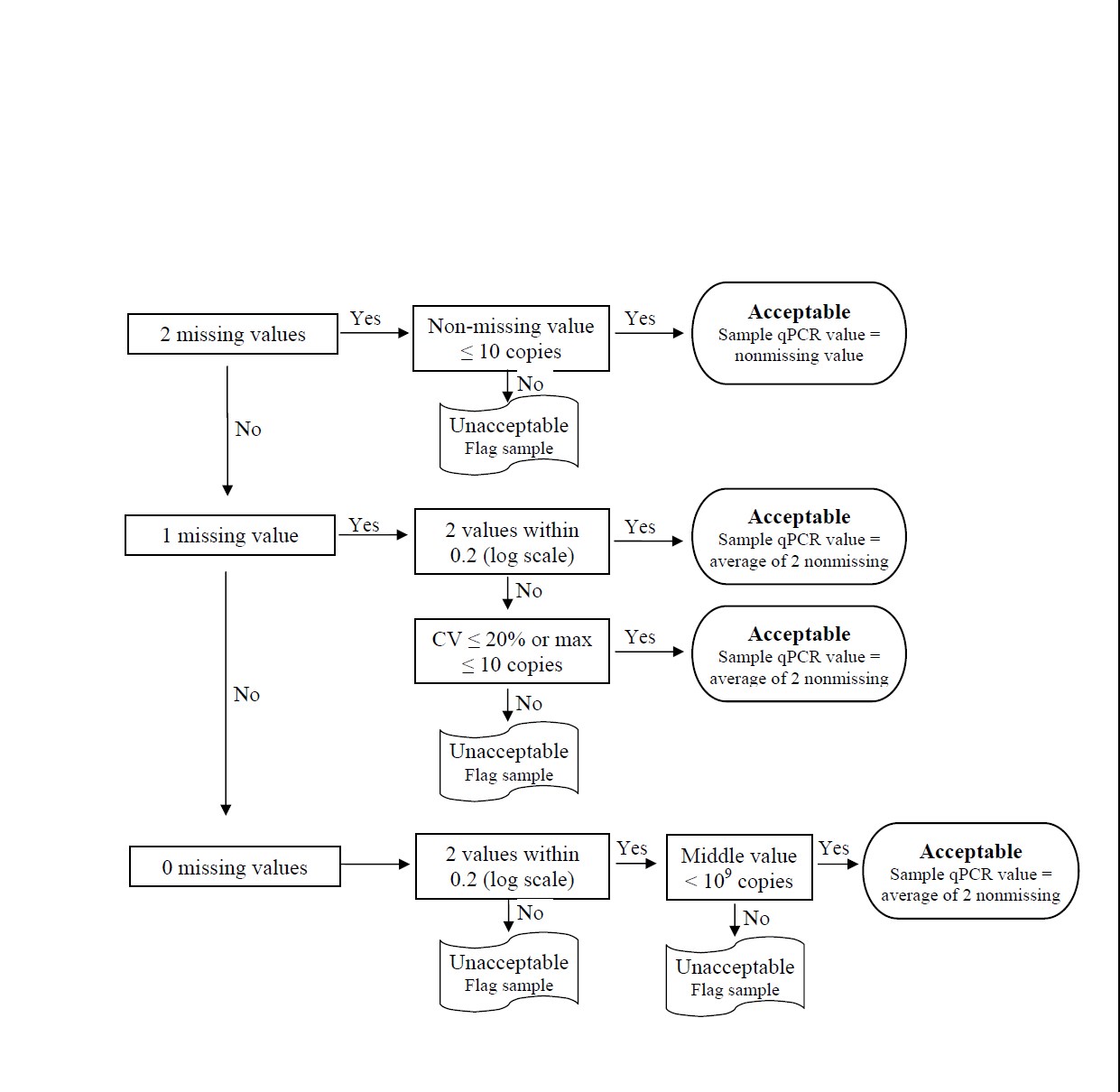

Supplement: Figure S1 — Quality control matrix with decision tree for samples with inconsistent results. There were 2,493 sample measurements (277 DNA samples x 9 qPCR assays) performed in triplicate. Coefficient of variation (CV) was calculated for each set of triplicate reactions. Results were considered consistent if all three values were not-detected, or if (1) all three replicates had detectable DNA, (2) the CV was less than 20%, and (3) the median value was less than 9. The upper limit of 109 was set to identify any reaction with copy numbers outside the range of the standard curve. For samples with consistent results, the mean value of the three replicates was taken to represent the copies of bacterial rRNA genes present in the sample. If all three replicates were not-detected, then the quantity of bacteria present was assumed to be below the limit of detection. Inconsistent results were examined in more detail and in most cases the result was determined using a set of quality control rules as follows: (1) if two values were not-detected and the third value was <10 copies/reaction, then the sample value equaled the detected value, and (2) if two non-missing values were within 0.2 log, with the third value being either missing or different by more than 0.2 log, then the sample value equaled the mean of two values. Assays that did not meet these criteria were considered unacceptable and qPCR reactions were rerun. (TIF) [file pone.0015101.s005.tif]

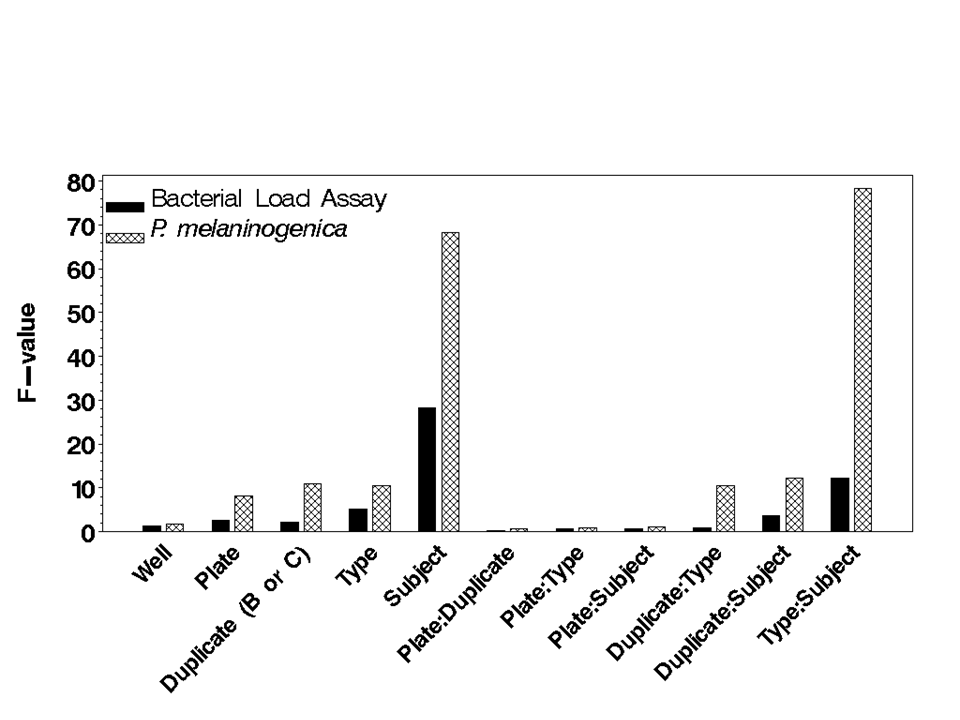

Supplement: Figure S2 — ANOVA analysis of total precision with between-run and within-run comparisons. Subject was the primary factor contributing to differences in measurement across plates and across wells (n = 24 samples). Five factors and six pairwise interactions were analyzed. There was greater variability across subjects for P. melaninogenica than for bacterial load assay. (TIF) [file pone.0015101.s006.tif]

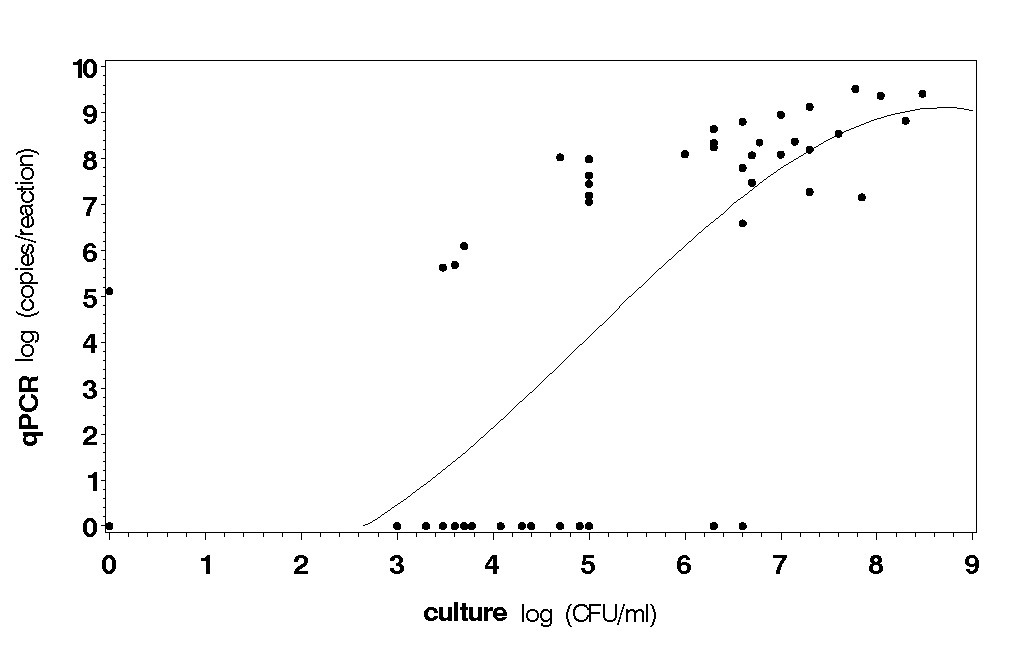

Supplement: Figure S3 — Sensitivity of S. aureus qPCR assay is highest for samples with quantities of S. aureus ≥105 cfu/ml by culture (n = 49 specimens). (TIF) [file pone.0015101.s008.tif]
